# An endoplasmic reticulum localized acetyl-CoA transporter is required for efficient fatty acid synthesis in *Toxoplasma gondii*

**DOI:** 10.1098/rsob.240184

**Published:** 2024-11-13

**Authors:** Biyun Qin, Bolin Fan, Yazhou Li, Yidan Wang, Bang Shen, Ningbo Xia

**Affiliations:** ^1^ National Key Laboratory of Agricultural Microbiology, College of Veterinary Medicine, Huazhong Agricultural University, Wuhan, Hubei Province, People’s Republic of China; ^2^ Key Laboratory of Preventive Veterinary Medicine in Hubei Province, Wuhan, Hubei Province, People’s Republic of China; ^3^ Hubei Hongshan Laboratory, Wuhan, Hubei Province, People’s Republic of China; ^4^ Shenzhen Institute of Nutrition and Health, Huazhong Agricultural University, Shenzhen, Guangdong Province, People’s Republic of China; ^5^ Shenzhen Branch, Guangdong Laboratory for Lingnan Modern Agriculture, Genome Analysis Laboratory of the Ministry of Agriculture, Agricultural Genomics Institute at Shenzhen, Chinese Academy of Agricultural Sciences, Shenzhen, Guangdong Province, People’s Republic of China; ^6^ College of Veterinary Medicine, South China Agricultural University, Guangzhou, Guangdong Province, People’s Republic of China

**Keywords:** apicomplexan, fatty acids, apicoplast, malaria, drug resistance

## Abstract

*Toxoplasma gondii* is an obligate intracellular parasite that can infect humans and diverse animals. Fatty acids are critical for the growth and proliferation of *T. gondii*, which has at least two pathways to synthesize fatty acids, including the type II de novo synthesis pathway in the apicoplast and the elongation pathway in the endoplasmic reticulum (ER). Acetyl-CoA is the key substrate for both fatty acid synthesis pathways. In the apicoplast, acetyl-CoA is mainly provided by the pyruvate dehydrogenase complex. However, how the ER acquires acetyl-CoA is not fully understood. Here, we identified a putative acetyl-CoA transporter (TgAT1) that localized to the ER of *T. gondii*. Deletion of TgAT1 impaired parasite growth and invasion *in vitro* and attenuated tachyzoite virulence *in vivo*. Metabolic tracing using ^13^C-acetate found that loss of TgAT1 reduced the incorporation of ^13^C into certain fatty acids, suggesting reduced activities of elongation. Truncation of AT1 was previously reported to confer resistance to the antimalarial compound GNF179 in *Plasmodium falciparum*. Interestingly, GNF179 had much weaker inhibitory effect on *Toxoplasma* than on *Plasmodium*. In addition, deletion of AT1 did not affect the susceptibility of *Toxoplasma* to GNF179, suggesting that this compound might be taken up differently or has different inhibitory mechanisms in these parasites. Together, our data show that TgAT1 has important roles for parasite growth and fatty acid synthesis, but its disruption does not confer GNF179 resistance in *T. gondii*.

## 1. Introduction

*Toxoplasma gondii* is an important zoonotic pathogen belonging to the phylum Apicomplexa, which contains a large group of unicellular parasites infecting humans and animals, including *Plasmodium* spp., *Cryptosporidium* spp. and *Eimeria* spp. that cause malaria, diarrhoea and coccidiosis, respectively [1–3]. *T. gondii* has an extremely wide host range and it is able to infect many warm-blooded animals [3]. A major question in the field is the metabolic strategy that enables *Toxoplasma* to survive in diverse hosts with distinct nutritional environments. It has been well demonstrated that *Toxoplasma* parasites can use multiple carbon sources to support their growth. Glucose and glutamine are preferred but neither one is essential [4,5]. Deletion of the main glucose transporter (GT1) that imports host and environmental glucose into the parasites only modestly reduced tachyzoite growth *in vitro* and had no impact on acute virulence *in vivo* [4]. Similar phenotypes were observed after deleting mitochondrial phosphoenolpyruvate carboxykinase (PEPCK_mt_), an enzyme that links the catabolism of glucose and glutamine [5]. Nonetheless, simultaneous inactivation of GT1 and PEPCK_mt_ caused severe growth inhibition [5], suggesting the metabolic cooperation of these two nutrients. In addition to glucose and glutamine, *T. gondii* parasites can also use lactate and even amino acids as alternative carbon sources to support parasite growth, as determined by the partial rescue of the pyruvate kinase 1 (PYK1) depletion mutant by lactate and alanine [6]. It has been postulated that the capacity to use diverse carbon sources may allow the parasites to establish infection in different environmental niches. In addition to synthesizing and generating metabolites by itself, *Toxoplasma* also has multiple pathways to scavenge nutrients and metabolites from host cells to support their propagation [4,7–12]. For example, it encodes a number of ApiATs (apicomplexan amino acid transporters) to import key amino acids like arginine, lysine [9,13] and tyrosine [11]. Furthermore, the dense granule proteins GRA17, GRA23, GRA47 and GRA72 can form pores in the parasitophorous vacuole (PV) membrane to facilitate the uptake of small molecules into PV from host cells [14,15]. *Toxoplasma* also has a micropore, which has been shown to have endocytosis functions, enabling the uptake of nutrients from host cells [8,16].

Metabolic flexibility in *T. gondii* is also reflected in fatty acid acquisition. *T. gondii* encodes genes to synthesize fatty acids, meanwhile it can also scavenge them from host cells [17–21]. Genomic analysis suggests that there are three possible pathways for fatty acid synthesis in *Toxoplasma*: the type I (FAS I) and type II (FAS II) fatty acid synthesis pathways, as well as the fatty acid elongation (FAE) pathway [19,22,23]. Among these, the biochemical activity of FAS I has not been demonstrated and remains to be determined. FAS II mainly contributes to the synthesis of medium-chain and long-chain saturated fatty acids, such as lipoic acid, myristic acid and palmitic acid [19,24–26]. All FAS II enzymes were found to be localized to the apicoplast, a non-photosynthetic plastid found in many apicomplexan parasites. Initially, the FAS II pathway was thought to be an ideal target for antiparasitic drug designs [22,24,27]. However, recent genetic studies argue against this idea. Deletion of *FabD* or *FabZ*, key enzymes in the FAS II pathway, resulted in viable and virulent *Toxoplasma* mutants [20], suggesting that FAS II is not essential for tachyzoite growth. Similarly, FAS II was also found to be dispensable for the asexual reproduction of blood-stage malaria parasites [28], although it has crucial roles at other developmental stages. Fatty acids synthesized in the apicoplast may be exported to the endoplasmic reticulum (ER) and are further elongated by the FAE pathway to produce very long-chain and unsaturated fatty acids [25]. The FAE pathway consists of three elongases (ELO-A, ELO-B and ELO-C), two reductases (enoyl-CoA reductase, ECR; keto-acyl-CoA reductase, KCR) and a dehydratase (hydroxyacyl-CoA dehydrogenase, DEH), all of which are localized in the ER [19]. Individual ablation of *ELO-A*, *ELO-B* or *ELO-C* did not obviously affect tachyzoite growth *in vitro*, although reduced synthesis of unsaturated fatty acids such as C26:1 was observed in the *ΔELO-B* and *ΔELO-C* mutants [29]. On the other hand, conditional knockdown of ECR or DEH resulted in strong defects in fatty acid synthesis, leading to severe growth inhibition of the parasites [19]. These results suggest that the FAE pathway is involved in the synthesis of fatty acid species that are key for tachyzoite growth. In addition to synthesis, *T. gondii* can also salvage host fatty acids to support parasite growth. It has been demonstrated that host cell lipid droplets (LDs) could be recruited to the vicinity of invaded *T. gondii* parasites. Staining human fibroblasts (HFF) infected with or without *T. gondii* by BODIPY 493/503 illustrated numerous LDs close to the PV of *T. gondii* in infected cells, whereas the LDs in uninfected cells were evenly distributed in the cytoplasm [30]. This phenomenon was more evident following the addition of oleic acid, which prompts host cells to generate copious amounts of LDs [31]. Within the PV, a parasite lipase discharges lipids from the host’s LDs, rendering them accessible to the parasite. On the other hand, host cells also evolved strategies to limit the uptake of host lipids by *T. gondii*, as a way to defend parasite infection [32]. For example, *Toxoplasma* infection triggers lipophagy in host cells, which helps the parasites to scavenge host fatty acids to support their proliferation [30]. As a response to infection, host cells induce mitochondrial fusion to limit the access of host fatty acids by *Toxoplasma* cells [32].

Both FAS II and FAE pathways require acetyl-CoA as a key substrate [19,20,28,29,33–35]. Previous studies have suggested that the apicoplast localized pyruvate dehydrogenase (PDH), which catalyses the conversion of pyruvate to acetyl-CoA, is the main source of acetyl-CoA in the apicoplast [36]. Knocking out the E1-alpha subunit of the PDH complex (*PDH-E1α*) caused slower growth of tachyzoites *in vitro*, which could be rescued by supplementation of exogenous myristic acid and palmitic acid [36]. Interestingly, mutants lacking PDH subunits were as virulent as the parental strain in mice, likely because the salvage of host fatty acids compensated the reduced fatty acid synthesis due to *PDH* deletion. While PDH is thought to be the main source of acetyl-CoA in the apicoplast, how acetyl-CoA is provided in the ER is still a mystery. It has been reported that acetate can be converted to acetyl-CoA in the cytosol of *T. gondii* by acetyl-CoA synthetase (ACS) [37]. The ^13^C-labelling experiments showed acetate-derived carbon could be incorporated into saturated and unsaturated fatty acids such as C20:0, C20:1, C22:1, C24:0, C24:1 and C26:1, which are mainly synthesized in the ER by FAE. In addition, knocking out the glucose transporter GT1 (*Δgt1*) resulted in defects in the synthesis of fatty acids such as C22:1 and C26:1, which could be rescued by exogenous acetate supplementation [18,38]. Accordingly, cytosolic acetyl-CoA may be produced by ATP citrate lyase (ACL) or ACS [39,40]. The deletion of either ACL or ACS alone did not affect parasite growth. However, the simultaneous deletion of both resulted in severe growth defects [39,40]. These results suggest that cytosolic acetyl-CoA can enter the ER. However, the underlying mechanism is not clear.

In human cells, an acetyl-CoA transporter (AT1) that imports cytosolic acetyl-CoA into the ER has been report [41]. AT1 is a transmembrane protein containing 11 or 12 transmembrane domains and biochemical studies have confirmed its localization in the ER membrane of Chinese hamster ovary (CHO) cells [41]. Using radioactive acetyl-CoA, it has been determined that the AT1 protein functions as an acyl-CoA transporter [41,42]. AT1 seems to be widely present in diverse organisms from bacteria to mammalian cells. *Toxoplasma* also encodes an AT1 orthologue. Endogenous tagging at the C-terminus suggested a perinuclear localization of AT1 in *T. gondii* [39]. These results indicate that AT1 may be the main source of acetyl-CoA in the ER. In this study, we found that AT1 is localized in the ER of *T. gondii*. Meanwhile, a genetic knockout strain (ME49*Δat1*) was constructed to assess its role in fatty acid synthesis and parasite growth.

## 2. Results

### 2.1. TgAT1 is localized to the endoplasmic reticulum

**Figure 1.**
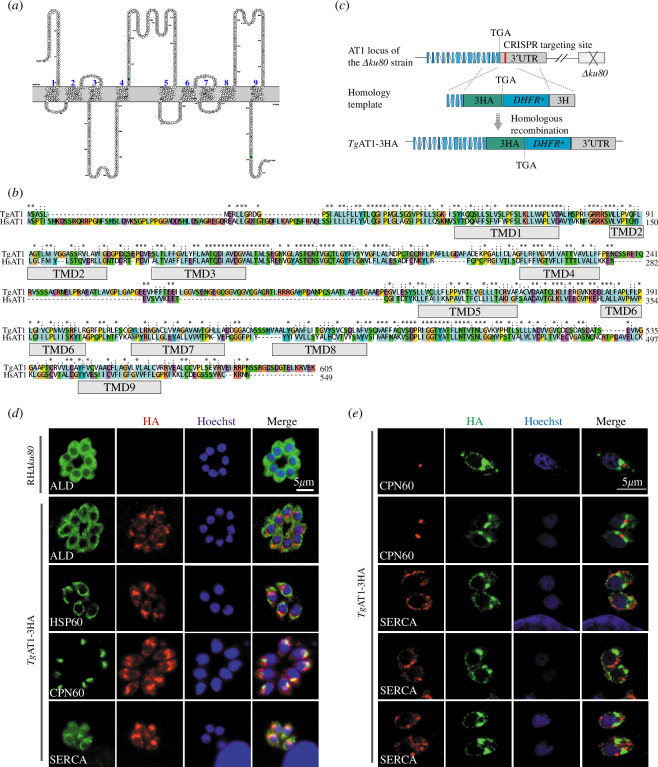
Subcellular localization of TgAT1 in *Toxoplasma* tachyzoites. (*a*) Predicted transmembrane topology of TgAT1. (*b*) Sequence alignment between TgAT1 and human AT1. The positions of the nine predicted transmembrane motifs are indicated as TMD1–TMD9. (*c*) Schematic diagram of the TgAT1-3HA strain construction using CRISPR/Cas9-mediated homologous recombination. Red bar represents the CRISPR/Cas9 targeting site. (*d*) Indirect immunofluorescence assays to detect the localization of TgAT1 by epifluorescence microscopy, using the TgAT1-3HA strain (probed with anti-HA). The cytoplasmic protein ALD, mitochondrial protein HSP60, apicoplast protein CPN60 and endoplasmic reticulum protein SERCA were included as co-localization references. (*e*) Confocal microscopy checking the co-localization of TgAT1-3HA with CPN60 or SERCA.

TgAT1 (TGME49_215940) was previously identified as a putative acetyl-CoA transporter in *T. gondii* [43]. Using the Phobius method that was integrated into the PROTTER tool [44], it was predicted that TgAT1 had nine transmembrane domains (figure 1*a*). When the amino acid sequence of TgAT1 was compared with that of human AT1, it was found that the two proteins shared 21.8% sequence similarity at full length level and much higher similarities in certain regions, which implies that TgAT1 may be a bona fide acetyl-CoA transporter (figure 1*b*).

To determine the localization of TgAT1 in *T. gondii*, we generated a stable strain of TgAT1-3HA by inserting a 3× HA tag to the C-terminus of endogenous TgAT1, using CRISPR/Cas9 assisted gene editing (figure 1*c*). Immunostaining analysis of the TgAT1-3HA strain using an HA antibody revealed that TgAT1 is distributed in the vicinity of the nucleus. To check where exactly TgAT1 is localized, co-localization studies of TgAT1-3HA with markers in different cellular compartments, including the cytoplasmic marker ALD, endoplasmic reticulum marker SERCA, mitochondrial marker HSP60 and apicoplast marker CPN60, were performed. The results indicate that TgAT1-3HA might have partial co-localization with HSP60 and CPN60 (figure 1*d*) when examined by epifluorescence microscopy. To more precisely identify the subcellular localization of TgAT1, confocal microscopy was used to compare the localization patterns of TgAT1-3HA with that of specific organellular markers. The results indicated that although the fluorescent signal of TgAT1-3HA was very close to that of CPN60, they did not overlap with each other (figure 1*e*), suggesting that TgAT1 is probably not located on or within the apicoplast. On the other hand, significant co-localization of TgAT1-3HA with the endoplasmic reticulum marker SERCA was observed (figure 1*e*). Interestingly, in these experiments, TgAT1-3HA was often found to accumulate in puncta structures in/on ER or other unknown structures very close to ER. Together, these results show that TgAT1-3HA is located in the ER and possibly other currently unknown structures. Previous studies also reported the perinuclear localization of TgAT1 that resembled the ER [39,45].

### 2.2. TgAT1 is required for tachyzoite invasion and growth

**Figure 2.**
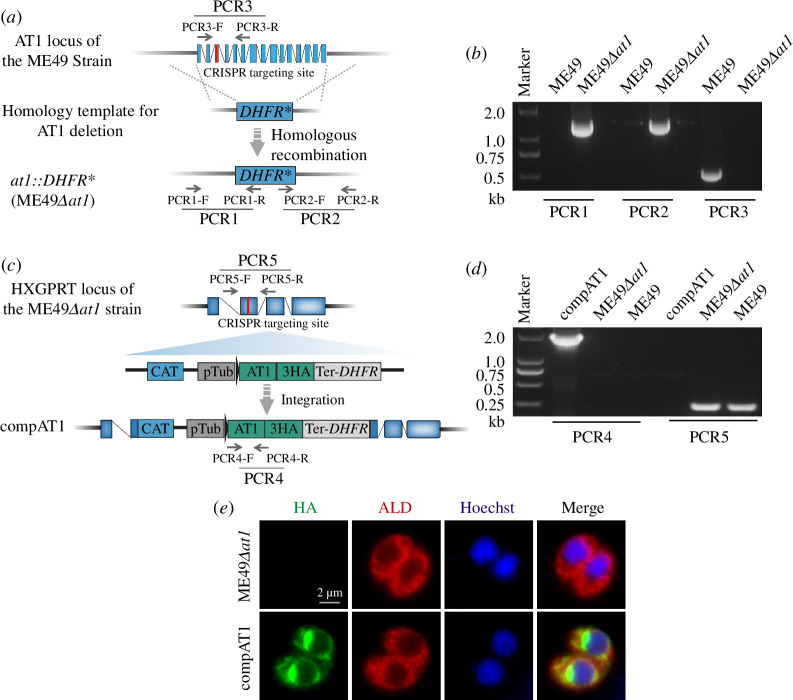
Construction of *TgAT1* knockout and complementing strains. (*a*) Schematic illustration of the construction of the ME49*Δat1* strain using the CRISPR/Cas9 gene editing technology. The red bar represents the CRISPR targeting site. (*b*) Diagnostic PCRs (PCR1, PCR2 and PCR3) used to identify the ME49*Δat1* mutant. (*c*) Diagram showing the strategy of inserting the AT1 complementing cassette into the *HXGPRT* locus of the ME49*Δat1* strain, using CRISPR/Cas9 gene editing technology. The red bar in *HXGPRT* represents the CRISPR targeting site. (*d*) PCR4 and PCR5 used to identify the compAT1 clone. (*e*) Indirect immunofluorescence was used to detect the expression of TgAT1-HA in the compAT1 strain. ME49*Δat1* was used as a control.

To investigate the biological functions of TgAT1 in *T. gondii*, we used CRISPR/Cas9 gene editing technology to replace the TgAT1 gene in the ME49 strain with the selection marker DHFR* to construct the *TgAT1* knockout strain (ME49*Δat1*) (figure 2*a*). After drug screening and limiting dilution, single clones were examined by diagnostic PCRs to screen for clean knockout strains (figure 2*b*). The presence of the PCR1 and PCR2 products indicated the correct integration of DHFR* into the *TgAT1* locus, whereas the absence of a PCR3 product confirmed the deletion of the *TgAT1* gene (figure 2*b*). To assist subsequent phenotypic analyses, we also constructed a complementation strain (compAT1) by introducing a *TgAT1* expressing cassette to the *HXGPRT* locus of ME49*Δat1* (figure 2*c,d*). The presence of a PCR4 product indicated the introduction of the *TgAT1* gene and the absence of a PCR5 product further confirmed the disruption of *HXGPRT* by insertion of *TgAT1* (figure 2*c,d*). Detection of HA tag expression in the compAT1 strain further confirmed the expression of TgAT1 in the compAT1 strain (figure 2*e*).

**Figure 3.**
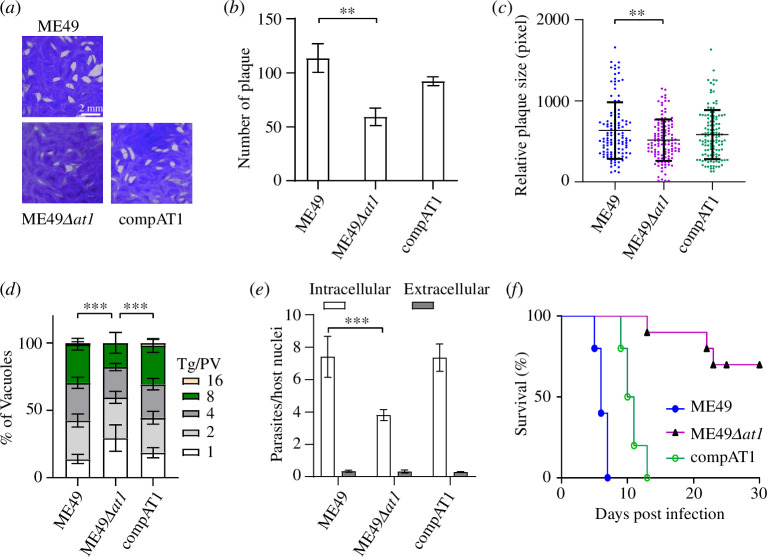
*TgAT1* knockout affects parasite invasion, growth and virulence. (*a*) Comparison of plaque formation between ME49, ME49*Δat1* and compAT1 strains. (*b*,*c*) Comparison of the relative sizes and number of plaques derived from (*a*). Values are means ± s.d. (**p < 0.01, Student’s *t*-test). (*d*) Comparison of parasite replication efficiency within HFF cells, as determined by the distribution of PVs containing 1, 2, 4, 8 or 16 tachyzoites. Data are means ± s.e.m. from three independent experiments (***p < 0.001, two-way ANOVA). (*e*) Invasion efficiency of parasites into HFF cells after 20 min of infection. Invasion efficiency was calculated as the number of invaded parasites divided by the number of host cell nuclei (means ± s.e.m., *n* = 3, ***p < 0.001, Student’s *t*-test). (*f*) Loss of *TgAT1* reduces parasite virulence *in vivo*. Survival curves of ICR mice (*n* = 10 per group) infected with tachyzoites of the indicated strains by intraperitoneal injection.

To investigate the role of TgAT1 in parasite growth *in vitro*, we first performed plaque assays to compare the overall fitness of the ME49*Δat1* mutant. The results show that ME49*Δat1* formed less (about 50%) plaques than the parental strain ME49 and the complementing strain compAT1 (figure 3*a,b*). The size of plaques formed by ME49*Δat1* strains was also slightly smaller than that of the ME49 or compAT1 strains (figure 3*c*). We also performed replication assay under standard growth conditions *in vitro* to compare their intracellular replication rates. The results suggest that the ME49*Δat1* parasites propagated more slowly (figure 3*d*). Compared with the wild-type strain ME49, the ME49*Δat1* mutant contained more PVs with 1 parasite but less PVs with 16 parasites, after 24 h replication (figure 3*d*). *TgAT1* complementation fully restored the replication defect. In addition, the ME49*Δat1* mutant also displayed significantly reduced invasion efficiency (figure 3*e*). Taken together, these data suggest that TgAT1 is important for *T. gondii* growth and invasion *in vitro*. Interestingly, deletion of *TgAT1* in the type I strain RH did not seem to affect parasite invasion during the lytic cycle [43]. The phenotypic differences between the type I versus type II *Δat1* mutants may stem from the different metabolic properties and requirements of the parental strains, which have intrinsic differences in recruiting host organelles like mitochondrion and then may have different capacities to scavenge host nutrients [46,47].

### 2.3. Deletion of *TgAT1* results in reduced parasite virulence

To assess the role of TgAT1 in parasite virulence *in vivo*, we infected ICR mice with ME49, ME49*Δat1* or compAT1 strains (100 parasites per mouse) by intraperitoneal injection and monitored their survival daily. Under the same conditions, ME49 and compAT1 parasites killed infected mice on days 7 and 13, respectively, while those infected with ME49*Δat1* had a survival rate of over 70% on day 30 (figure 3*f*). The above result showed that deletion of *TgAT1* in the ME49 strain reduced the death of infected mice. On the other hand, *TgAT1* complementation largely restored the virulence of the ME49*Δat1* mutant (figure 3*f*), suggesting that TgAT1 contributes to parasite virulence in *T. gondii*.

### 2.4. TgAT1 contributes to fatty acids synthesis in the parasite

**Figure 4.**
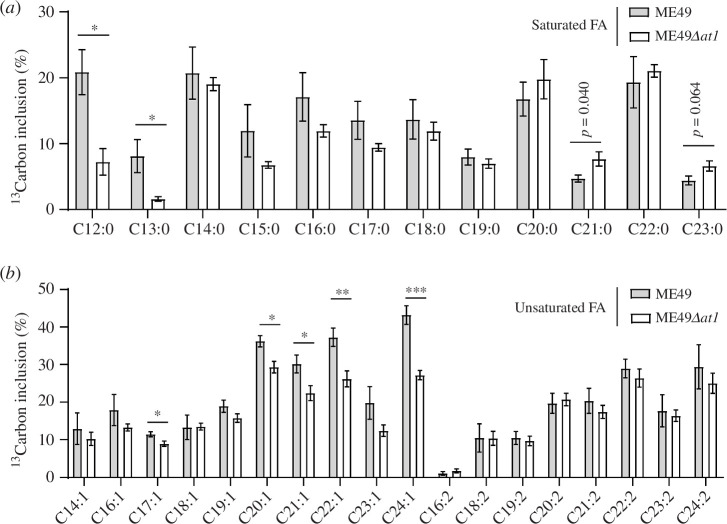
*TgAT1* deletion results in reduced fatty acid synthesis in *Toxoplasma gondii*. Intracellular ME49 and ME49 *Δat1* tachyzoites were treated with ^13^C-labelled acetate for 48 h. The incorporation of ^13^C into saturated (*a*) and unsaturated (*b*) fatty acids was measured by GC–MS and plotted separately. Means ± s.e.m., *n* = 5, *p < 0.05, **p < 0.01, ***p < 0.001, Student’s *t*-test.

To investigate whether TgAT1 is involved in fatty acid synthesis in *T. gondii*, intracellular parasites of the ME49 and ME49*Δat1* strains were labelled with 8 mM ^13^C-acetate for 48 h, which could be taken up by tachyzoites and converted to ^13^C‐acetyl-CoA [35,38]. Fatty acids from purified ME49 and ME49*Δat1* tachyzoites (3 × 10^7^) were extracted with chromatographic grade methanol and trichloromethane. GC–MS was then used to determine the effect of *TgAT1* deletion on parasite fatty acid abundance. The saturated fatty acids C12:0 to C16:0 represent fatty acids synthesized by FAS II in the apicoplast. The metabolic labelling results show that incorporation of ^13^C into the two main fatty acids synthesized in the apicoplast, C14:0 and C16:0, was not significantly affected. However, the labelling of C12:0 and C13:0 was reduced upon TgAT1 depletion (figure 4*a*). The ME49*Δat1* parasites showed decreased ^13^C labelling of C17:1, C20:1, C21:1, C22:1 and C24:1 (figure 4*b*), which were mainly produced in the ER by FAE. These results suggest that *TgAT1* deletion leads to reduced fatty acid synthesis in the parasites. It is worth noting that TgAT1 deletion did not completely abolish the incorporation of ^13^C into fatty acids. This is probably due to the fact that fatty acid synthesis involves more than one organelle. Although reduced acetyl-CoA supply in the ER caused by TgAT1 deletion is expected to decrease fatty acid synthesis by FAE, nonetheless, before reaching ER for elongation, fatty acids are first synthesized in the apicoplast by the de novo synthesis pathways FAS II, which should not be completely inhibited by TgAT1 deletion.

### 2.5. TgAT1 deletion does not confer resistance to the GNF179 compound that inhibits *Plasmodium* growth

**Figure 5.**
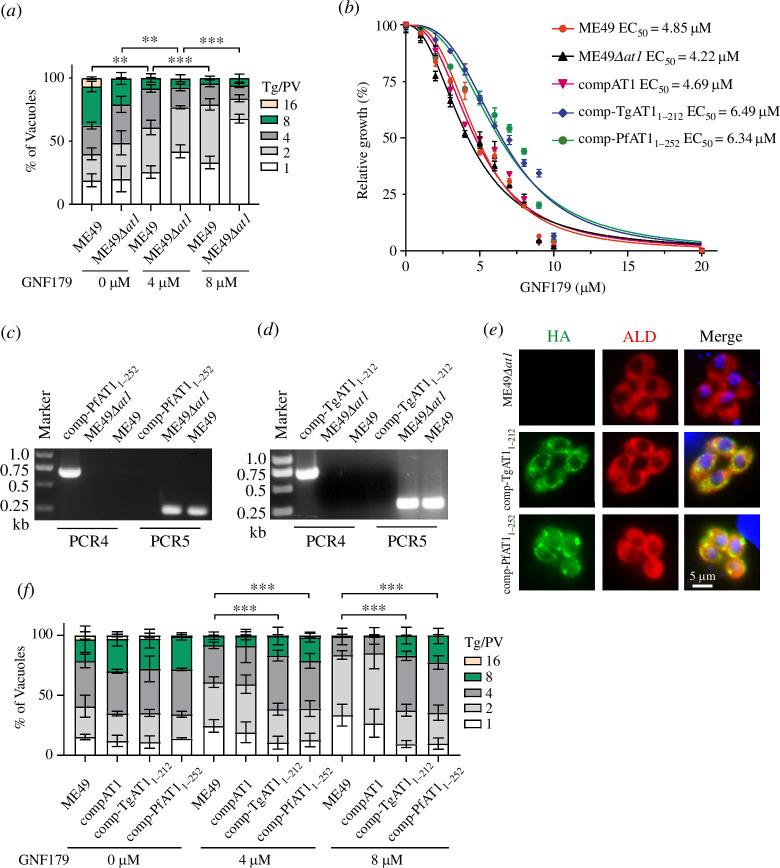
The relationship between TgAT1 and parasite drug resistance. (*a*) Replication efficiency of indicated strains in the presence of different concentrations of GNF179 (**p < 0.01, ***p < 0.001, two-way ANOVA). (*b*) Determination of EC_50_ of GNF179 on indicated *T. gondii* strains. All EC_50_ values are presented as the means ± s.e.m. (*n* = 3) of three biological replicates. (*c*,*d*) Diagnostic PCRs identified the clonal strains comp-PfAT1_1-252_ and comp-TgAT1_1-212_. (*e*) Expression of PfAT1_1-252_ and TgAT1_1-212_, as shown by IFA using mouse anti-HA. The ME49*Δat1* strain was used as a control. (*f*) Replication efficiency of indicated strains treated with 0 µM, 4 µM or 8 µM GNF179 for 30 h. ***p < 0.001, two-way ANOVA.

It has been reported that the imidazolopiperazine compound GNF179 could efficiently block the growth of *Plasmodium falciparum* parasites at low nanomolar concentrations [48]. Mutations in PfAT1, including nonsense mutations at S242 or L253 residues that resulted in truncations upstream of the fifth transmembrane domain (TM) of the 10 TM bearing PfAT1 protein, caused resistance to GNF179, increasing the EC_50_ of GNF179 over 350-fold [48,49]. It was believed that PfAT1 is involved in taking up GNF179 to its target site. Therefore, its disruption leads to drug resistance. To investigate whether GNF179 has inhibitory effects on *T. gondii* growth, we performed replication assays using the ME49 and ME49*Δat1* strains at different GNF179 concentrations. As the concentration of GNF179 increased, the replication rate of wild-type strain ME49 or knockout strain ME49*Δat1* gradually decreased (figure 5*a*). This result indicates that GNF179 could inhibit the growth of *T. gondii*. However, the concentration required to inhibit *Toxoplasma* growth is much (approx. 500 times) higher than that used to block the growth of *Plasmodium*. In addition, deletion of *TgAT1* did not seem to affect the susceptibility of *Toxoplasma* parasites to GNF179 (figure 5*a*). To more precisely measure the effect of GNF179 on different *Toxoplasma* parasites, half-maximal effective concentration (EC_50_) was determined using the replication assay. The results showed that the EC_50_ values for the ME49, ME49*Δat1* and compAT1 strains were 4.85 μM, 4.22 μM and 4.69 μM, respectively (figure 5*b*), suggesting that TgAT1 deletion indeed did not have a significant impact on GNF179 susceptibility, which is in sharp contrast to the *Plasmodium* case.

Termination of PfAT1 at leucine 253 was shown to confer resistance to the inhibitory effect of GNF179 on *Plasmodium* growth [48]. PfAT1 belongs to the MFS family secondary transporter with ten transmembrane domains and termination at leucine 253 removed six of them [48]. Therefore, such premature termination probably abolished the function of this protein entirely. Nonetheless, there is still a possibility that the truncated AT1 may still be functional but with altered substrate specificity, pumping GNF179 away from the site of action. In this case, deletion of AT1 would not confer resistance but expression of truncated AT1 might, since the truncated AT1 maintains an intact MFS domain. To test this possibility, PfAT1_1-252_ or the equivalent truncation TgAT1_1-212_ were expressed from the *HXGPRT* locus of the ME49*Δat1* strain to construct the comp-PfAT1_1-252_ and comp-TgAT1_1-212_ strains, respectively (figure 5*c,d*), using the same strategy as illustrated in figure 2*c*. Diagnostic PCRs confirmed the correct integration of PfAT1_1-252_ (figure 5*c*) and TgAT1_1-212_ cassettes (figure 5*d*). The expression of PfAT1_1-252_ and TgAT1_1-212_ was further confirmed by immunofluorescent staining (figure 5*e*). The overall distribution of PfAT1_1-252_ and TgAT1_1-212_ in the parasites is similar to that of the full-length TgAT1, but they were more dispersed (figure 5*e*). Replication assays using the ME49, compAT1, comp-PfAT1_1-252_ and comp-TgAT1_1-212_ strains cultured in the presence of different concentrations of GNF179 for 30 h showed that expression of truncated AT1s slightly altered the susceptibility of parasites to GNF179 (figure 5*f*). Without GNF179 treatment, these strains replicated at very similar rates. However, in the presence of 4 μM GNF179, the percentage of parasitophorous vacuoles (PVs) containing four and eight tachyzoites in ME49 and compAT1 strains was only 40%, compared with 60% in the comp-PfAT1_1-252_ and comp-TgAT1_1-212_ strains (figure 5*f*). At the concentration of 8 μM GNF179, approximately 80% of the PVs formed by ME49 and compAT1 strains contained two and four tachyzoites, and no PV reached the size of eight tachyzoites. However, in the comp-PfAT1_1-252_ and comp-TgAT1_1-212_ strains, around 20% of the PVs contained eight tachyzoites (figure 5*f*). Similarly, the EC_50_ values of GNF179 on the comp-PfAT1_1-252_ and comp-TgAT1_1-212_ strains were 6.49 μM and 6.34 μM, respectively (figure 5*b*), which is slightly higher than that on ME49 (4.85 μM) (figure 5*b*). Together, these results suggested that truncated TgAT1 or PfAT1 indeed very mildly decreased the susceptibility of parasites to GNF179, although the mechanisms are unknown.

## 3. Discussion

As an obligate intracellular pathogen that infects diverse types of hosts and host cells, *Toxoplasma gondii* is able to acquire fatty acids by multiple approaches, including uptake of fatty acids from host cells, de novo synthesis in the apicoplast, and elongation in the ER [17–19,21,50]. While the biological significance of each of these pathways has been examined, one question left unaddressed is how the synthetic pathways acquire the key substrate acetyl-CoA. As a molecule with key roles in metabolism and protein modification, acetyl-CoA could be generated in multiple organelles in *Toxoplasma* parasites, including the mitochondrion, cytosol and apicoplast [36,39,40,51]. PDH in the apicoplast that catalyses the conversion of pyruvate to acetyl-CoA was thought to be the main source of acetyl-CoA for de novo fatty acid synthesis in the apicoplast [36]. On the other hand, the mechanism of acetyl-CoA acquisition in the ER is completely unknown. In this study, we identified a potential acetyl-CoA transporter, TgAT1, which localizes to the ER of *Toxoplasma* tachyzoites. A *TgAT1* deletion strain was successfully constructed, suggesting that it is not essential for tachyzoite growth or survival. However, the ME49*Δat1* mutants did show slower growth *in vitro* and reduced virulence *in vivo*, suggesting important functions of TgAT1 for optimal parasite propagation. Moreover, *TgAT1* deleted mutants exhibited decreased efficiency of incorporating cytosolic acetyl-CoA into fatty acids that are synthesized in the ER, indicating that TgAT1 is indeed involved in the import of acetyl-CoA into ER to fuel fatty acid synthesis.

**Figure 6.**
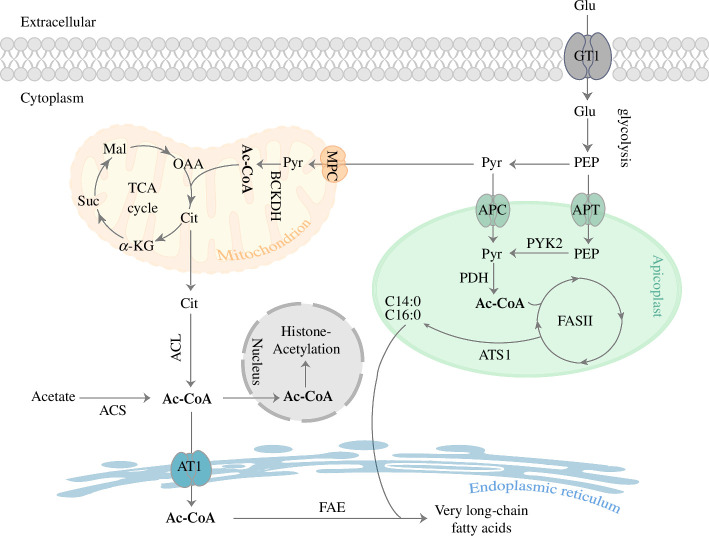
A model for the functions and acquisition pathways of acetyl-CoA in multiple organelles of *Toxoplasma. Toxoplasma* has multiple ways to produce and use acetyl-CoA in different organelles. It can be generated from pyruvate by BCKDH or PDH in the mitochondrion and apicoplast, respectively, which is then used to fuel the TCA cycle in the mitochondrion, or the FAS II pathway for fatty acid synthesis in the apicoplast. Acetyl-CoA can also be generated by ACS or ACL in the cytosol, using acetate or citrate as substrates, respectively. In the nucleus, acetyl-CoA may be generated by ACS, or it is imported from the cytosol, to enable protein acetylation. Acetyl-CoA is also a critical substrate for fatty acid elongation pathway FAE in the ER, but no acetyl-CoA synthesis has been reported in the ER. By contrast, the TgAT1 transporter is probably involved in translocating cytosolic acetyl-CoA to the ER. Glu, glucose; GT1, glucose transporter; PEP, phosphoenolpyruvate; Pyr, pyruvate; APC, apicoplast pyruvate carrier; FAS II, type II fatty acid synthesis pathway; APT, apicoplast phosphate translocator; PYK2, pyruvate kinase 2; PDH, pyruvate dehydrogenase; ATS1, apicoplast glycerol-3-phosphate acyltransferase; TCA cycle, tricarboxylic acid cycle; MPC, mitochondrial pyruvate carrier; BCKDH, branched-chain α-ketoacid dehydrogenase; Ac-CoA, acetyl-CoA; Cit, citrate; α-KG, α-ketoglutarate; Suc, succinate; Mal, malate; ACS, acetyl-CoA synthetase; ACL, ATP citrate lyase; FAE, fatty acid elongation pathway; AT1, acetyl-CoA transporter. The arrows represent catalytic reactions or pathways (it should be noted that this is a highly simplified outline and that the number of arrows does not reflect the number of enzymatic steps required).

As a metabolite with key functions in multiple organelles, acetyl-CoA can be generated by multiple pathways in *T. gondii* parasites. In the apicoplast, acetyl-CoA is needed for de novo fatty acid synthesis by the FAS II pathway and it is mainly provided by the PDH complex (figure 6). Deletion of any one of the PDH subunits leads to slower growth of tachyzoites and this growth deficiency could be largely rescued by the supplementation of C14:0 and C16:0, suggesting that PDH is indeed an important source of acetyl-CoA for the apicoplast [36]. Interestingly, the phenotypes of the *PDH* deletion mutants are very similar to that of mutants lacking *FabD* or *FabZ*, enzymes involved in the initiation and elongation phases of the FAS II pathway, respectively [20]. These results may indicate that PDH is the major source of acetyl-CoA for the apicoplast. Furthermore, we have recently identified a novel apicoplast pyruvate carrier (APC), which transports cytosolic pyruvate into the apicoplast to fuel acetyl-CoA production. Deletion of APC results in reduced activities of FAS II and impaired integrity of the apicoplast organelle, leading to parasite growth arrest [52]. In the mitochondria, acetyl-CoA is required for TCA cycle and is mainly produced by the branched-chain α-ketoacid dehydrogenase (BCKDH), which catalyses the conversion of pyruvate imported by mitochondrial pyruvate carrier (MPC) into acetyl-CoA (figure 6). Tachyzoites lacking MPC or BCKDH-E1α reduced the incorporation of glucose-derived carbons into acetyl-CoA and displayed significant growth defects [34,51]. For the organelles that need acetyl-CoA but do not have synthetic pathways, import from the cytosol is a feasible approach. The two enzymes that produce acetyl-CoA in the cytosol, acetyl-CoA synthetase (ACS) and ATP citrate lyase (ACL), are synthetic lethal. Mutants lacking both enzymes experienced reduced fatty acid elongation, hypo-acetylation of nucleo-cytosolic proteins and dramatic changes in gene expression [40], suggesting that ER and the nucleus acquire their acetyl-CoA from the cytosol. Here we found that TgAT1 is mainly responsible for importing acetyl-CoA into the ER (figure 6). Consistent with this notion, AT1 is localized in the ER of *T. gondii*, human cells and other model eukaryotes. It seems like the ER localization is highly conserved across species. Loss of AT1 in *Toxoplasma* caused a mild growth defect. Meanwhile, it led to reduced fatty acid synthesis in the parasites, as indicated by the metabolic flux measurements. Although we cannot completely rule out the possibility that the reduced fatty acid synthesis in the ME49*Δat1* mutant is a general consequence of slow parasite growth, our results rather suggest a model that AT1 transports cytoplasmic acetyl-CoA into the ER to fuel fatty acid elongation, which is critical for *Toxoplasma* growth [19]. Therefore, reduced fatty acid synthesis in the ME49*Δat1* mutant is probably responsible for its decreased growth and proliferation. While *Toxoplasma* cells are known to be able to salvage fatty acids and lipids from host cells [17–19,30,32,53–55], importance of ECR or DEH that are involved in the FAE pathway, as well as the roles of TgAT1 described here, indicated that there are likely fatty acid species that cannot be scavenged from the host and must be synthesized by the parasites. On the other hand, mutants lacking ECR or DEH had much stronger growth defects than the ME49*Δat1* mutants, suggesting that there may be additional pathways for acetyl-CoA supply in the ER of *Toxoplasma* parasites, which deserve further investigations.

AT1 belongs to the secondary transporter family protein MFS26, which is associated with resistance to imidazolopiperazine [48,56–59]. Previous studies have demonstrated that mutations in PfAT1 could lead to resistance to the antimalarial drug chloroquine and other unrelated scaffolds [48]. In this study, we found that the concentrations required to inhibit the growth of WT *Toxoplasma* (around 4.8 μM) and WT *Plasmodium* (5–9 nM) are dramatically different. In addition, in *Plasmodium*, truncation of AT1 increased the EC_50_ of GNF179 by more than 350-fold, changing it from low nM to mM. This can be easily explained by the hypothesis that AT1 imports GNF179 to the ER [48], where its bona fide target is located. By contrast, in *Toxoplasma*, deletion of AT1 did not significantly change the susceptibility of parasites to GNF179, suggesting that TgAT1 is not the primary target for GNF179 within the *Toxoplasma* cell. What is more, the EC_50_ of GNF179 on AT1 truncated *Plasmodium* mutants was comparable with that on WT *Toxoplasma*, indicating that *Toxoplasma* is naturally resistant to GNF179 at concentrations that kills *Plasmodium*. There are several possibilities for this difference, for example: firstly, different permeability of this compound into the parasites; secondly, *Toxoplasma* may not have the same target as *Plasmodium* for GNF179. On the other hand, GNF179 at mllimolar concentrations does inhibit the growth of *Toxoplasma*. Plus, expression of the truncated AT1 in the ME49*Δat1* mutant did slightly increase the EC_50_ of GNF179 (from 4.8 μM to 6.5 μM), indicating mild resistance. It is hypothesized that truncated TgAT1 may lose its function but remain stable and still be expressed in the ER membrane (as indicated by IFA results in figure 5*e*). This may result in the occupation of space in the ER membrane, which could affect the exchange of substances between ER and the cytosol. On the other hand, the truncated AT1 mutants contained an MFS domain. We speculate that the MFS domain is a functional transporter, which may pump the GNF179 compounds out of the space where the target is located. Further studies are needed to figure out these possibilities.

## 4. Material and methods

### 4.1. Biological resources

The ME49 strain of *T. gondii* and its derivative strains were propagated in human foreskin fibroblasts (ATCC no. SCRC-1041, USA), which were cultured in Dulbecco’s modified Eagle’s medium (DMEM) supplemented with 10% fetal bovine serum (Life Technologies, USA), 100 µg ml^−1^ streptomycin and 10 mM L-glutamine [60].

### 4.2. Plasmids construction

All primers and plasmids used are listed in electronic supplementary material, tables S1 and S2. Site-specific CRISPR plasmids were generated by replacing the *UPRT*-targeting gRNA in pSAG1-Cas9-U6-sg*UPRT* with a gRNA targeting *Tg*AT1. Homologous templates for gene replacement were constructed by multi-fragment cloning using the ClonExpress MultiS cloning kit (Vazyme Biotech, Nanjing, China). The homology template pAT1::DHFR* used for *Tg*AT1 replacement was constructed by cloning the 5′ and 3′ homologous arms (approximately 1 kb) amplified from the genomic DNA of ME49 and the pyrimethamine resistant cassette DHFR* amplified from pUPRT-DHFR*-D into the *pUC19* vector. The *Tg*AT1 coding sequence and AT1_1-212_ used for pComp-AT1 and pComp-AT1_1-212_ were amplified from the cDNA of ME49. Subsequently, these fragments were cloned into pCom-LDH1 to replace the coding sequence of LDH1 [61]. The pComp-PfAT1_1-252_ was constructed in a similar way, except that the coding sequence of PfAT1_1-252_ was codon optimized and synthesized by Sangon Biotech (Shanghai, China).

### 4.3. Construction of AT1 deletion and complementation mutants

All transgenic parasites used in this study are listed in electronic supplementary material, table S3. To create the *TgAT1* deletion mutant in ME49, a total of 7500 ng of CRISPR/Cas9 plasmid targeting AT1 and 1500 ng of homologous template (AT1::DHFR*) were co-transfected into fresh ME49 parasites. Pyrimethamine at final concentration of 1 μM was used for drug selection and single clones were obtained by limited dilution in a 96-well plate containing HFF monolayers. The construction of the complemented strains involved co-transfecting 7500 ng of CRISPR/Cas9 plasmid targeting *HXGPRT* and 1500 ng of complementation fragment into ME49*Δat1*. All single clones were confirmed by PCR and further validated by IFA before use.

### 4.4. Indirect immunofluorescence staining

Fresh-egressed parasites were used to infect HFF monolayers. After 24 h of infection, cells were fixed with 4% paraformaldehyde for 15 min and permeabilized with 0.1% Triton X-100 for 15 min. Blocking was performed with 10% fetal bovine serum for 30 min. Primary and secondary antibodies were diluted in 1% BSA-PBS and incubated at 37°C for 20 min. Hoechst was used as a staining reagent to observe the host and parasite nucleus. The antibodies used in the assay include mouse anti-HA (MBL, 1:1000), goat anti-rabbit/rat Alexa Fluor 594 (Invitrogen, 1:1000), goat anti-rabbit/rat Alexa Fluor 488 (Invitrogen, 1:1000), rabbit anti-mouse Alexa Fluor 594 (Invitrogen, 1:1000), rabbit anti-mouse Alexa Fluor 488 (Invitrogen, 1:1000), and Hochest 33342 (Beyotime, 1:1000), rabbit anti-ALD (provided by Dr David Sibley at Washington University School of Medicine, produced using AA 1-497 of TGGT1_236040 as antigen and used at 1:1000 dilution for IFA) [62], rabbit anti-SERCA (prepared by our laboratory, produced using AA 373-805 of TGGT1_230420 as antigen and used at 1:500 dilution for IFA) [63], rabbit anti-HSP60 (prepared by our laboratory, produced using AA 439-1143 of TGGT1_247550 as antigen and used at 1:1000 dilution for IFA) [64], and rabbit anti-CPN60 (provided by Dr Jia Honglin at Harbin Veterinary Research Institute, produced using AA 1258-2001 of TGGT1_240600 as antigen and used at 1:1000 dilution for IFA) [65]. The samples were examined using a BX53 Olympus microscope (Olympus Life Science, Tokyo, Japan) or a Zeiss LSM 880 confocal laser microscope (Zeiss, Germany) equipped with narrow band pass filters (Narrow UV filter with 360–370 nm excitation and 420–460 nm emission for Hochest 33342; Narrow Blue filter with 470–495 nm excitation and 510–550 nm emission for Alexa Fluor 488; RFP Filter with 545–580 nm excitation and >610 nm emission for Alexa Fluor 594). Fluorescent images were acquired with the same settings (such as exposure time, transmission intensity, etc.) in the same experiment.

### 4.5. Plaque assays

Tachyzoites were purified by passing them through a 3.0 μm membrane filter and counted using a haemocytometer under a Nikon Eclipse TS100 phase contrast microscope (Nikon Instruments, Tokyo, Japan). Two hundred parasites were added to each well of a six-well plate containing HFF monolayers and incubated at 37°C with 5% CO_2_ for 10 days. The cells were then fixed with polyformaldehyde and stained with crystal violet (0.1%). Images of the plaques were captured using a scanner to analyse the number and relative size of the plaques. All strains were tested independently three or more times with three internal replicates each time.

### 4.6. Virulence assays

Seven-week-old female ICR mice were each infected with 100 tachyzoites of the ME49, ME49*Δat1* and compAT1 by intraperitoneal injection. Clinical signs were recorded daily, and the cumulative mortality rate was plotted as a Kaplan–Meier survival curve.

### 4.7. Invasion assays

Freshly egressed tachyzoites (5 × 10^6^) were added to a monolayer of HFF cells on coverslips and allowed to invade for 20 min at 37°C. The coverslips were then quickly washed with PBS and fixed with 4% paraformaldehyde for 15 min. Extracellular parasites were stained with pig anti-*Toxoplasma* antibodies. Then, the samples were permeabilized with 0.1% Triton X-100 for 15 min and stained with rabbit anti-*Toxoplasma* antibodies to stain all intracellular and extracellular parasites. Secondary antibodies, FITC-conjugated goat anti-pig IgG and Alexa 594-conjugated goat anti-rabbit IgG, were used to detect the primary antibodies. The coverslips were then examined using fluorescence microscopy. The invasion efficiency was calculated as the number of invaded parasites divided by the number of host cell nuclei.

### 4.8. Replication assays

Intracellular replication assays that determined the proliferation rates of strains were preformed following previously described protocols [66]. The invaded tachyzoites were cultured under normal conditions for 24 h. The replication efficiency of indicated strains treated with 0 µM, 4 µM, or 8 µM GNF179 for 30 h. The samples were then fixed, permeabilized and stained for immunofluorescence detection (as described in the previous section). Only vacuoles stained in red were examined, and the number and size of the vacuoles containing 1, 2, 4, 8 and 16 parasites were counted and analysed. At least 150 PVs were examined for each strain in each experiment, and each strain was tested independently three times. Similarly, the EC_50_ values for GNF179 on different strains were also determined by replication assays. Different concentrations of GNF179 (0, 1, 2, 3, 4, 5, 6, 7, 8, 9, 10 and 20 μM) were used to treat freshly invaded, intracellular parasites for 24 h and then the average number of parasites in each PV was determined to calculate the relative growth rates of strains. The EC_50_ values were obtained by curve fitting using the ‘dose–response–inhibition’ regression program in GraphPad Prism 9.0 software.

### 4.9. Metabolic analysis

For fatty acid analysis, the parasites were cultured for 48 h in a medium containing 8 mM ^13^C-acetate (Sigma Aldrich). After filtration, the samples (3 × 10^7^ parasites) were added to the mixed chromatographic organic solvent chloroform : methanol (2 : 1) according to the ratio of organic phase to water phase 4 : 1, and the operation was repeated three times after complete extraction and centrifugation at 3000 r.p.m. for 20 min. The organic phase (lower layer) was transferred to a new glass tube using a glass microinjector, dried with nitrogen to approximately 1 ml, vortexed and further dried to a transparent film without heating during the drying process. The long chain fatty acids were separated and quantified by C8 reversed phase liquid chromatography-electrospray coupled to high resolution mass spectrometry. The metabolomics dataset used in this study is listed in electronic supplementary material, table S4.

### 4.10. Data analysis

All statistical analyses were performed using Prism 9.0 software (GraphPad Software, Inc., La Jolla, CA, USA). All experiments requiring statistical analysis were performed at least three times and results were presented as mean ± s.d. or s.e.m. (as indicated in figure legends). Replication experiments were analysed using two-way ANOVA; survival curves were analysed using Gehan–Breslow–Wilcoxon test; and all other experiments were analysed using Student’s *t*-test.

## Data Availability

All data generated in this study are included in the paper and its electronic supplementary material files [67].
